# Predictive value of the serum sodium to log(D-dimer) ratio for the risk of all-cause death in patients with chronic heart failure with different ejection fractions

**DOI:** 10.1186/s12872-026-05609-y

**Published:** 2026-07-20

**Authors:** Yujuan Peng, Wei Zhang, Yunhong Yang, Tao Shi, Fazhi Yang, Sirui Yang, Xinuo Ma, Ping Xia, Lixing Chen

**Affiliations:** 1 Department of Cardiology, The First Affiliated Hospital of Kunming Medical University, Kunming, China; 2 Emergency Department, The First Affiliated Hospital of Kunming Medical University, Kunming, China

**Keywords:** Serum sodium, D-dimer, Heart failure, Prognosis, Ejection fraction, All-cause mortality

## Abstract

**Background:**

The ratio of serum sodium to log(D-dimer) (log-SDR) in different types of chronic heart failure (CHF) is not well established.

**Methods:**

A total of 1,221 hospitalized CHF patients from the First Affiliated Hospital of Kunming Medical University between January 2017 and October 2021 were retrospectively analyzed. Patients were categorized into log-SDR-L group (log-SDR < 47.90) and log-SDR-H group (log-SDR ≥ 47.90).Prognostic assessments included Kaplan-Meier survival analysis, Cox survival analyses and time-dependent Receiver operating characteristic (ROC) curves analysis to evaluate predictive performance.

**Results:**

We collected data from 1008 patients with CHF. Kaplan–Meier survival analysis revealed that patients with high log-SDR levels had better overall survival (OS). After multivariate adjustment Cox proportional hazards analysis, the level of log-SDR was still independently related to mortality, regardless of CHF subtype.

**Conclusions:**

log-SDR is an important predictor of all-cause mortality in patients with HF, especially female HFrEF plus HFmrEF(HR:0.927, 95%CI:0.898–0.958, *p* < 0.001). Lower log-SDR levels are associated with an increased risk of all-cause mortality, irrespective of the HF subtype.

**Supplementary Information:**

The online version contains supplementary material available at 10.1186/s12872-026-05609-y.

## Introduction

Chronic heart failure (CHF) represents the end stage of various cardiovascular diseases [1]. Characterized by high rates of hospital readmission and mortality, CHF has emerged as a major global public health challenge [2]. Data from the Global Burden of Disease (GBD) study indicate a consistent increase in the prevalence of heart failure (HF) across all sociodemographic index levels from 1990 to 2021, underscoring its substantial societal impact [3]. Although the incidence of HF in high-income countries has plateaued or declined over the past decade, its prevalence continues to rise, largely due to population aging, growing risk factor burden, and improved survival attributable to advances in therapy [4]. Consequently, identifying clinically applicable biomarkers to aid in the risk stratification and management of patients with acute exacerbations of chronic heart failure (AECHF) is of critical importance.

Hyponatremia, a common electrolyte disturbance, has been associated with prognosis in HF. This association may be mediated by water and sodium retention secondary to cardiac dysfunction, while high-dose diuretic therapy can lead to excessive sodium loss, both contributing to reduced serum sodium levels [5, 6]. Evidence suggests that correction of hyponatremia is correlated with improved outcomes in HF patients, whereas persistent or severe hyponatremia is linked to increased mortality and worse prognosis [7]. D-dimer, a specific degradation product of cross-linked fibrin, serves as a biomarker reflecting the balance between thrombosis and fibrinolysis, and is widely used to assess activation of the coagulation system [8]. Elevated D-dimer levels are frequently observed in cardiovascular diseases and have been significantly correlated with adverse long-term outcomes in CHF [9, 10]. In patients with acute left ventricular failure, increased D-dimer levels are also closely associated with short-term adverse events during hospitalization [11].

The sodium-to-D-dimer ratio (SDR) has recently emerged as a novel composite prognostic marker, demonstrating initial utility in predicting treatment response and outcomes in patients with advanced gastric cancer receiving first-line chemotherapy [12]. Similar to malignancies, CHF is characterized by systemic inflammation, electrolyte disturbances, and a hypercoagulable state [1]. Given the established prognostic relevance of both serum sodium and D-dimer in HF, combining these markers into the SDR is pathophysiologically grounded rather than merely a mathematical convenience. Hyponatremia reflects neurohormonal activation and volume dysregulation—central mechanisms in HF progression—whereas elevated D-dimer indicates activation of the thrombosis–inflammation axis, a key driver of adverse outcomes. Critically, these pathways interact synergistically rather than in isolation [1, 13]. As a ratio, the SDR quantitatively captures the imbalance between these two interconnected processes. However, because serum sodium follows a relatively narrow distribution and D-dimer a markedly skewed one, we log-transformed D-dimer to reduce potential distortion from its extreme values. The resulting ratio, defined as log-SDR, represents serum sodium divided by log-transformed D-dimer. To our knowledge, no prior studies have evaluated the association between log-SDR and clinical outcomes in HF, and its prognostic role across phenotypic subgroups remains unexplored.

Left ventricular ejection fraction (LVEF) remains the most commonly used parameter for assessing systolic function in HF. According to LVEF, HF is categorized into three subtypes: heart failure with reduced ejection fraction (HFrEF, LVEF ≤ 40%), heart failure with mildly reduced ejection fraction (HFmrEF, LVEF 41%–49%), and heart failure with preserved ejection fraction (HFpEF, LVEF ≥ 50%). This classification is clinically relevant, as these subtypes differ in etiology, patient demographics, comorbidity profiles, and treatment responses [14]. Therefore, this study aims to evaluate the impact of log-SDR on clinical outcomes in HF patients with different ejection fractions.

## Methods

### Study population

We conducted a retrospective analysis of 1,221 medical records from patients diagnosed with AECHF at the First Affiliated Hospital of Kunming Medical University between January 2017 and October 2021. The inclusion criteria were a diagnosis of AECHF with New York Heart Association (NYHA) functional class III or IV and/or a brain natriuretic peptide (BNP) level ≥ 500 pg/mL. Patients were excluded if they had missing serum sodium or D-dimer data, comorbid malignancies, hematologic diseases, severe renal (defined as creatinine clearance ≤ 20%) or hepatic dysfunction (defined as Child-Pugh class C), or incomplete follow-up data. After applying these criteria, 1,008 patients were included in the final analysis.

### Data collection and definitions

Comprehensive demographic information, medical history, clinical data, and laboratory parameters were collected from the electronic medical records of all enrolled patients. Laboratory assessments comprised a complete blood count, tests of liver and renal function, a lipid profile, electrolyte levels, and electrocardiography. Blood samples were drawn after a 12-hour fasting period, typically on the morning following admission. All samples were promptly transported to the central laboratory of the First Affiliated Hospital of Kunming Medical University and analyzed according to established procedures. The primary study endpoint was all-cause mortality. Survival status and the date of death were ascertained via structured telephone follow-up with patients or their family members. The log-SDR was calculated as serum sodium (mmol/L) divided by log(D-dimer)(ng/ml). Subsequently, patients were stratified into two groups based on the optimal cut-off of log-SDR value: a low log-SDR group (log-SDR-L, log-SDR < 47.90; *n* = 412) and a high log-SDR group (log-SDR-H, log-SDR ≥ 47.90; *n* = 596).

### Statistical analysis

Continuous variables are expressed as mean ± standard deviation for normally distributed data or median with interquartile range (IQR; 25th–75th percentiles) for non-normally distributed data. Due to a skewed distribution, BNP levels were logarithmically transformed prior to analysis. Group comparisons were performed using independent-samples t-tests or Mann–Whitney U tests for continuous variables, as appropriate, and Pearson’s chi-square tests for categorical variables.Survival analysis was conducted using Kaplan–Meier curves, with between-group differences assessed by the log-rank test. To identify predictors of all-cause mortality, univariate Cox regression analyses were first performed. Variables showing a significant association (*p* < 0.05) in the univariate analysis were subsequently entered into a multivariable Cox proportional hazards model. The prognostic value of log-SDR, treated as a continuous variable, was evaluated using both univariate and multivariable Cox regression models. Results are reported as hazard ratios (HRs) with corresponding 95% confidence intervals (CIs). The predictive performance of log-SDR for all-cause mortality was further evaluated using time-dependent receiver operating characteristic (ROC) curves, and the area under the curve (AUC) was calculated. Statistical analyses were performed using SPSS 27.0 software, and a p-value < 0.05 was considered statistically significant.

## Results

### Baseline patient characteristics

After applying exclusion criteria for in-hospital mortality, missing data on electrolytes, coagulation, fibrinolysis, or LVEF, and loss to follow-up, 1,008 patients with CHF were included in the final analysis. The cohort had a mean age of 66.9 years, and 61.3% were male, comprising 588 patients with HFrEF or HFmrEF and 420 patients with HFpEF. Compared to the log-SDR-H group, the log-SDR-L group had a higher proportion of New York Heart Association (NYHA) class IV and a higher rate of diuretic therapy. Additionally, the log-SDR-L group exhibited higher levels of log-transformed B-type natriuretic peptide (log-BNP), aspartate aminotransferase (AST), blood urea nitrogen (BUN), and white blood cell count (WBC). No significant differences were observed in albumin (ALB) or fibrinogen (Fib) levels between the two groups (Table 1).

**Table 1:** Baseline characteristics according to log-SDR level

Variables	Total *n* = 1008	log-SDR-L *n* = 412	log-SDR-H *n* = 596	*P*-value
Basic characteristics				
Age, years	66.88 ± 12.44	68.66 ± 13.18	65.68 ± 11.92	0.109
Sex, female %	390(38.7)	162(39.3)	228(38.3)	0.733
BMI, kg/㎡	22.98 ± 3.84	22.84 ± 3.90	23.15 ± 3.81	0.843
DBP, mmHg	75.88 ± 15.25	74.97 ± 14.08	76.49 ± 15.79	0.003
NYHA Ⅳ, %	372(36.9)	189(45.9)	183(30.7)	< 0.001
Aetiologies, n (%)				
Hypertension, %	557(55.3)	232(56.3)	325(54.5)	0.576
Atrial fibrillation, %	339(33.6)	137(33.3)	202(33.9)	0.833
Coronary disease, %	529(52.5)	214(51.9)	315(52.9)	0.776
CRT/CRTD, %	91(9.0)	35(8.5)	56(9.4)	0.624
Prior medication use, n (%)				
Diuretics, %	767(76.1)	354(85.9)	413(69.3)	< 0.001
ACEI/ARB/ARNI, %	578(57.3)	236(57.3)	342(57.4)	0.076
β-receptor blockers, %	618(61.3)	225(54.6)	393(65.9)	0.023
SGLT-2%	245(24.3)	118(28.6)	127(21.3)	0.273
Laboratory indicators				
WBC, 10^12/L	6.92(5.57,8.98)	7.35(5.89,10.03)	6.72(5.47,8.41)	< 0.001
NEU, 10^9/L	4.48(3.49,6.26)	4.99(3.76,7.20)	4.37(3.38,5.90)	< 0.001
LYM, 10^9/L	1.40(1.03,1.85)	1.29(0.96,1.71)	1.47(1.08,1.89)	< 0.001
Potassium, mmol/L	3.94 ± 0.60	3.99 ± 0.65	3.92 ± 0.57	0.004
Sodium, mmol/L	141.06 ± 4.32	139.64 ± 4.51	141.85 ± 3.98	0.004
LogBNP	3.16 ± 0.28	3.25 ± 0.29	3.10 ± 0.26	0.002
AST, U/L	27.85(20.00,42.18)	31.60(21.63,51.50)	26.35(19.40,38.95)	< 0.001
GLB, g/L	31.24 ± 5.84	32.22 ± 6.15	30.67 ± 5.63	0.106
ALB, g/L	36.72 ± 4.46	35.67 ± 4.36	37.46 ± 4.39	0.599
BUN, mmol/L	7.33(5.65,10.26)	7.92(6.06,11.99)	6.91(5.47,9.44)	< 0.001
Cr, µmol/L	103.00(81.90,134.10)	111.20(86.45,147.55)	98.80(80.13,124.78)	< 0.001
Fib, g/L	3.40(2.73,4.16)	3.53(2.75,4.36)	3.36(2.71,4.12)	0.056
Log(D-dimer)	2.8 ± 0.50	3.24 ± 0.25	2.5 ± 0.38	< 0.001
HDL-C, mmol/L	0.98(0.81,1.18)	0.94(0.76,1.17)	1.01(0.85,1.20)	< 0.001
LDL-C, mmol/L	2.15(1.64,2.75)	2.03(1.56,2.67)	2.25(1.66,2.86)	< 0.001
log-SDR	52.15 ± 11.06	43.33 ± 3.37	58.24 ± 10.41	< 0.001

According to log-SDR level, the patients were divided into two groups: log-SDR-L: log-SDR < 47.90, log-SDR-H: log-SDR ≥ 47.90. Differences in normally distributed continuous variables were compared using an independent sample t test, and those in nonnormally distributed data were compared using the Mann-Whitney U rank sum test. The χ2 test was used to compare between-group differences in categorical variables. P values are derived from comparing the log-SDR-L group with the log-SDR-H group. A P value < 0.05 was considered indicative of statistical significance

*BMI* body mass index, *DBP* diastolic blood pressure, *NYHA* New York Heart Association, *CRT* cardiac resynchronisation therapy, *CRTD* Cardiac Resynchronisation Therapy Defbrillato, *ACEI* angiotensin-converting enzyme inhibitor, *ARB* angiotensin receptor blocker, *ARNI* angiotensin receptor neprilysin inhibitor, *SGLT-2I* sodium-glucose cotransporter 2 inhibitor, *WBC* white blood cell, *NEU* neutrophil, *LYM* lymphocyte, *BNP* brain natriuretic peptide, *AST* aspartate aminotransferase, *GLB* globulin, *ALB* albumin, *BUN* blood urea nitrogen, *Cr* creatinine, Fib fibrinogen,*HDL-C* high density lipoprotein cholesterol, *LDL-C* low density lipoprotein cholesterol, *log-SDR* sodium to log(D-dimer) ratio

### Survival analyses based on log-SDR

**Fig. 1:**
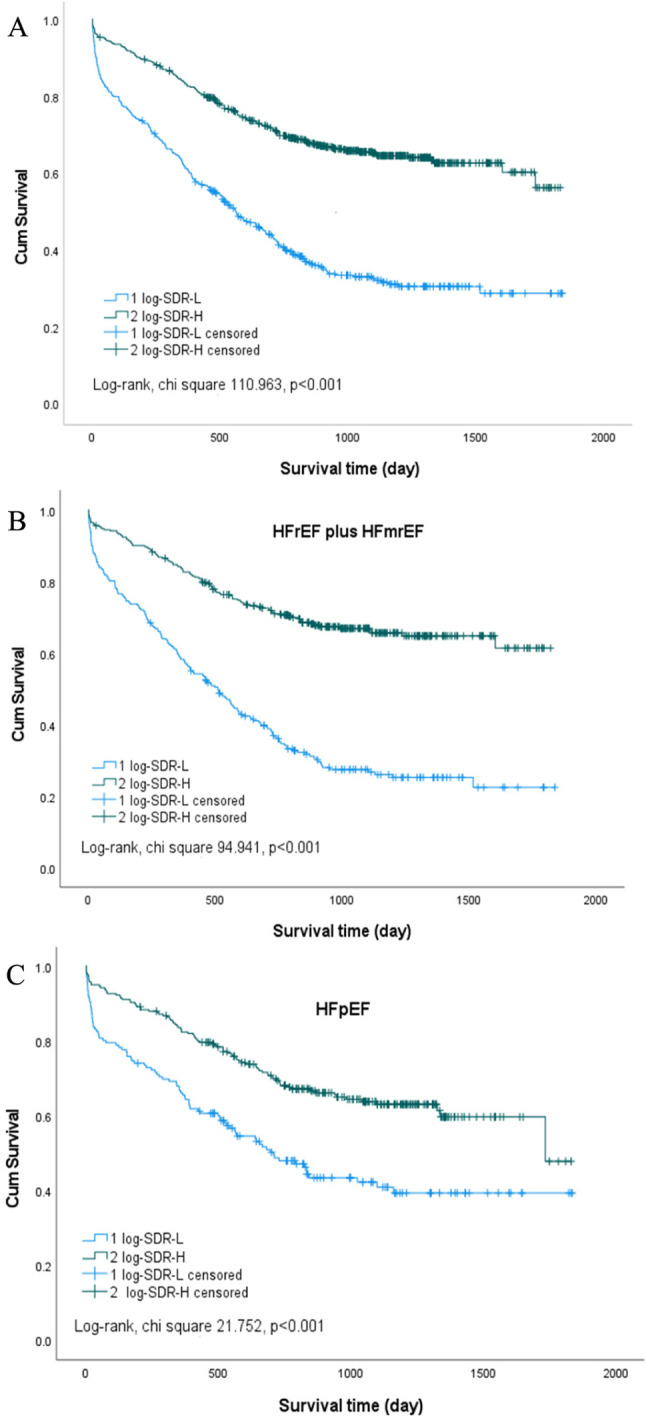
Kaplan‒Meier survival curves according to the optimal cut-off value of the log-SDR for all patients (**A**), HFrEF plus HFmrEF (**B**) and HFpEF (**C**)

Kaplan–Meier survival analysis was performed to evaluate the effect of log-SDR on patient prognosis. The log-SDR-H group was significantly associated with better overall survival (OS) in all patients (log-rank test, chi-square = 110.963, *p* < 0.001) (Fig. 1A), in those with HFrEF plus HFmrEF (log-rank test, chi-square = 94.941, *p* < 0.001) (Fig. 1B), and in those with HFpEF (log-rank test, chi-square = 21.752, *p* < 0.001) (Fig. 1C).

### log-SDR as an independent predictor

In univariate Cox proportional hazard analysis, log-SDR was significantly associated with all-cause mortality in all CHF patients. After adjusting for age, gender, systolic blood pressure(SBP), NYHA functional class, LogBNP, WBC, haemoglobin(HGB), globulin(GLB), unconjugated bilirubin(UCB), cardiac troponin(CTn), total cholesterol(TC), estimated glomerular filtration rate(eGFR), multivariate Cox proportional hazard analysis confirmed that log-SDR remained an independent predictor of all-cause mortality (HR0.965, 95% CI 0.953–0.977, *p* < 0.001). This association persisted in both HFrEF plus HFmrEF and HFpEF subgroups (HFrEF plus HFmrEF: HR0.949, 95% CI 0.932–0.967, *p* < 0.001; HFpEF: HR0.980, 95% CI 0.964–0.997, *p* = 0.021) (Table 2).

**Table 2:** Univariate and multivariate Cox analysis of log-SDR for predicting all-cause mortality in HF patients

All Patients				
	Univariable		Multivariable	
	HR(95%)	*P*-value	HR(95%)	*P*-value
Age	1.030(1.022,1.039)	< 0.001	1.014(1.005,1.024)	0.004
Female	Reference			
Male	1.022(0.849,1.231)	0.818		
SBP	0.992(0.988,0.996)	< 0.001	0.992(0.988,0.997)	< 0.001
NYHAIII	Reference			
NYHAIV	2.416(2.016,2.896)	< 0.001	1.961(1.613,2.385)	< 0.001
WBC	1.046(1.024,1.069)	< 0.001		
HGB	0.991(0.987,0.994)	< 0.001	0.993(0.989,0.997)	0.002
GLB	1.008(0.993,1.024)	0.305	0.974(0.958,0.990)	0.002
UCB	1.000(0.989,1.012)	0.959		
CTn	1.041(1.027,1.055)	< 0.001	1.022(1.006,1.038)	0.006
LogBNP	6.118(4.370,8.565)	< 0.001	3.048(2.130,4.362)	< 0.001
TC	0.826(0.746,0.913)	< 0.001		
eGFR	0.975(0.970,0.981)	< 0.001	0.986(0.980,0.993)	< 0.001
log-SDR	0.947(0.937,0.958)	< 0.001	0.965(0.953,0.977)	< 0.001

HFrEF plus HFmrEF				
	Univariable		Multivariable	
	HR(95%)	*P*-value	HR(95%)	*P*-value
Age	1.025(1.014,1.035)	< 0.001		
Female	Reference			
Male	0.947(0.744,1.206)	0.659		
SBP	0.990(0.985,0.996)	< 0.001	0.989(0.983,0.995)	< 0.001
NYHAIII	Reference	< 0.001		
NYHAIV	2.376(1.883,2.999)	< 0.001	1.699(1.319,2.188)	< 0.001
WBC	1.054(1.025,1.083)	< 0.001		
HGB	0.990(0.985,0.995)	< 0.001	0.992(0.986,0.997)	0.002
GLB	1.010(0.990,1.030)	0.323	0.968(0.948,0.988)	0.002
UCB	1.002(0.989,1.015)	0.789		
CTn	1.040(1.019,1.062)	< 0.001	1.036(1.010,1.063)	0.006
LogBNP	8.170(5.050,13.219)	< 0.001	3.467(2.013,5.974)	< 0.001
TC	0.803(0.705,0.914)	< 0.001		
eGFR	0.972(0.965,0.979)	< 0.001	0.981(0.972,0.990)	< 0.001
log-SDR	0.929(0.913,0.944)	< 0.001	0.949(0.932,0.967)	< 0.001

HFpEF				
	Univariable		Multivariable	
	HR(95%)	*P*-value	HR(95%)	*P*-value
Age	1.048(1.033,1.063)	< 0.001	1.044(1.026,1.062)	< 0.001
Female	Reference			
Male	1.093(0.815,1.467)	0.552		
SBP	0.996(0.989,1.002)	0.197		
NYHAIII	Reference			
NYHAIV	2.432(1.814,3.259)	< 0.001	2.906(2.107,4.007)	< 0.001
WBC	1.037(1.002,1.073)	0.036		
HGB	0.990(0.985,0.996)	0.001		
GLB	1.007(0.981,1.033)	0.594		
UCB	0.995(0.973,1.016)	0.631		
CTn	1.043(1.025,1.061)	< 0.001		
LogBNP	6.168(3.628,10.485)	< 0.001	3.959(2.238,7.003)	< 0.001
TC	0.849(0.720,1.000)	0.051		
eGFR	0.978(0.969,0.0.986)	< 0.001		
log-SDR	0.967(0.952,0.982)	< 0.001	0.980(0.964,0.997)	0.021

Only variables retaining independent statistical significance (*p* < 0.05) in the final multivariable model are shown in the “multivariate analysis” column.

*SBP* systolic blood pressure, *NYHA* New York Heart Association, *WBC* white blood cell, *HGB* haemoglobin, *GLB* globulin, *UCB* unconjugated bilirubin, *cTn *cardiac troponin, *BNP *brain natriuretic peptide, *TC *total cholesterol, *eGFR* estimated glomerular filtration rate, *log-SDR *sodium to log(D-dimer) ratio

A significant association was observed between log-SDR and all-cause mortality when patients were stratified by sex and HF subtype. Following multivariable adjustment, each unit increase in log-SDR as a continuous variable was associated with a greater reduction in mortality risk in female patients compared with male patients overall. Furthermore, irrespective of sex, the protective effect of log-SDR was more pronounced in HFrEF plus HFmrEF patients than in HFpEF patients. Specifically, among female patients with HFrEF plus HFmrEF, each unit increase in log-SDR was associated with an approximately 7.3% reduction in all-cause mortality risk (HR: 0.927, 95% CI: 0.898–0.958). In contrast, the corresponding risk reductionamong female HFpEF patients was 2.5% (HR: 0.975, 95% CI: 0.947–0.991). In male patients, the magnitude of risk reduction was comparatively smaller: a 3.8% decrease was observed in male HFrEF plus HFmrEF patients (HR: 0.962, 95% CI: 0.941–0.984), and a 1.7% decrease in male HFpEF patients (HR: 0.983, 95% CI: 0.963–0.998) (Table 3).

### Predictive ability of log-SDR

**Fig. 2:**
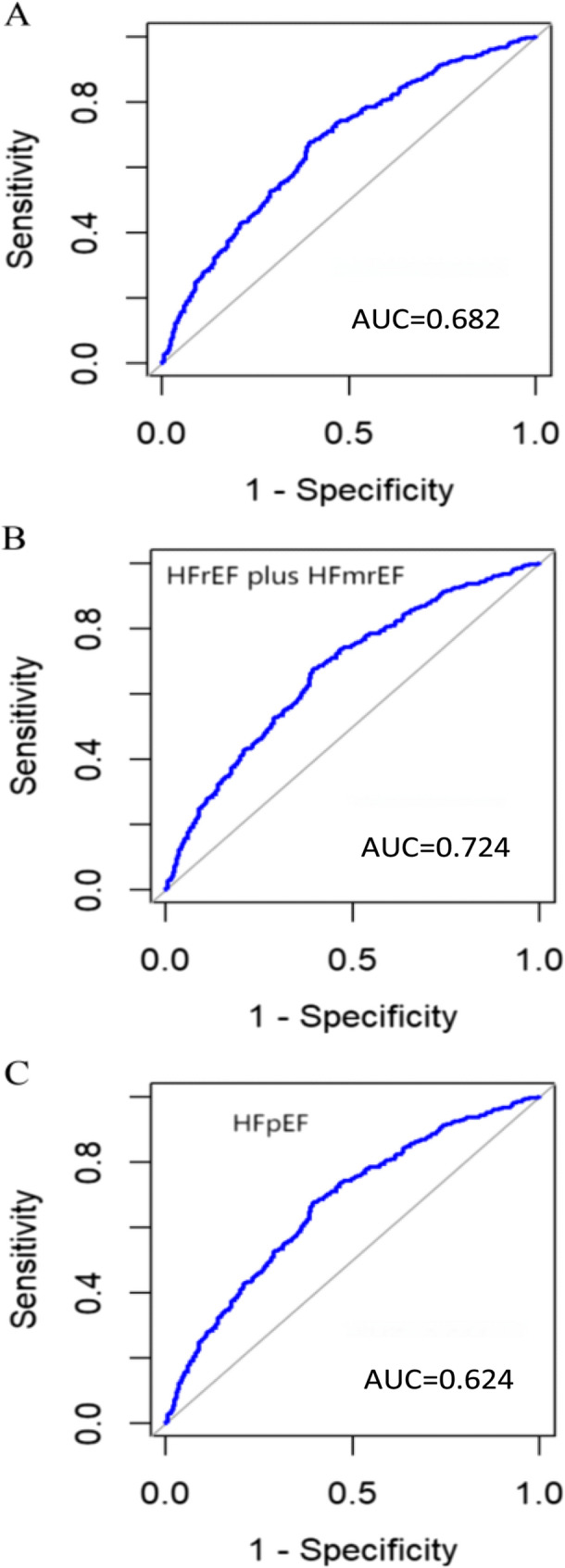
Time-dependent receiver operating characteristic (ROC) curves of log-SDR with reference line for all patients (**A**), HFrEF plus HFmrEF (**B**), and HFpEF (**C**)

ROC curves were plotted to evaluate the prognostic value of log-SDR, and the corresponding AUC values were calculated. Among patients with HFrEF plus HFmrEF, the area under the curve was 0.724. Among patients with HFpEF, the area under the curve was 0.624 (Fig. 2).

**Table 3:** Univariate and multivariate Cox model analysis of all-cause mortality in patients with HF based on gender subgroups

Female								
	HFrEF plus HFmrEF				HFpEF			
	Univariable		Multivariable		Univariable		Multivariable	
	HR(95%)	*P*-value	HR(95%)	*P*-value	HR(95%)	*P*-value	HR(95%)	*P*-value
Age	1.023(1.006,1.040)	0.009			1.054(1.029,1.078)	< 0.001	1.046(1.017,1.076)	0.002
SBP	0.989(0.980,0.999)	0.026	0.988(0.978,0.999)	0.034	1.004(0.995,1.014)	0.388		
NYHAIII	Reference							
NYHAIV	2.076(1.406,3.065)	< 0.001	1.675(1.090,2.576)	0.019	2.312(1.474,3.626)	< 0.001	3.147(1.844,5.372)	< 0.001
WBC	1.050(1.004,1.098)	0.034	1.061(1.003,1.123)	0.040	1.039(0.962,1.121)	0.33	1.079(1.007,1.156)	0.031
HGB	0.990(0.982,0.998)	0.017			0.983(0.973,0.993)	< 0.001		
GLB	1.006(0.976,1.037)	0.702	0.971(0.943,1.000)	0.049	1.021(0.981,1.063)	0.308		
UCB	0.980(0.950,1.011)	0.201	0.960(0.925,0.996)	0.028	0.980(0.946,1.016)	0.267		
CTn	1.004(0.961,1.049)	0.856			1.026(0.986,1.068)	0.207		
LogBNP	10.585(4.784,23.423)	< 0.001	2.530(1.020,6.275)	0.045	6.440(2.982,13.998)	< 0.001	3.578(1.461,8.767)	0.005
TC	0.728(0.587,0.903)	0.004			0.904(0.714,1.146)	0.404		
eGFR	0.975(0.964,0.986)	< 0.001			0.975(0.963,0.988)	< 0.001		
log-SDR	0.910(0.883,0.937)	< 0.001	0.927(0.898,0.958)	< 0.001	0.956(0.933,0.979)	< 0.001	0.975(0.947,0.991)	0.032

Male								
	HFrEF plus HFmrEF				HFpEF			
	Univariable		Multivariable		Univariable		Multivariable	
	HR(95%)	*P*-value	HR(95%)	*P*-value	HR(95%)	*P*-value	HR(95%)	*P*-value
Age	1.025(1.013,1.038)	< 0.001			1.045(1.026,1.065)		1.042(1.020,1.065)	< 0.001
SBP	0.991(0.984,0.998)	0.008	0.990(0.982,0.997)	0.007	0.989(0.981,0.998)	0.018		
NYHAIII	Reference							
NYHAIV	2.553(1.910,3.412)	< 0.001	1.848(1.337,2.555)	< 0.001	2.510(1.704,3.697)	< 0.001	2.712(1.769,4.157)	< 0.001
WBC	1.057(1.021,1.094)	0.002			1.037(0.997,1.097)	0.07		
HGB	0.989(0.983,0.985)	< 0.001	0.989(0.982,0.996)	0.003	0.994(0.987,1.001)	0.112		
GLB	1.012(0.986,1.039)	0.386	0.959(0.931,0.989)	0.008	0.997(0.965,1.031)	0.877		
UCB	1.007(0.994,1.019)	0.281			1.005(0.977,1.034)	0.718		
CTn	1.057(1.033,1.081)	< 0.001	1.050(1.021,1.081)	< 0.001	1.050(1.028,1.071)	< 0.001	1.028(1.000,1.057)	0.049
LogBNP	7.120(3.890,13.034)	< 0.001	4.005(1.995,8.039)	< 0.001	5.984(2.882,12.426)	< 0.001	5.088(2.341,11.056)	< 0.001
TC	0.850(0.722,1.002)	0.052			0.808(0.644,1.014)	0.066		
eGFR	0.970(0.961,0.980)	< 0.001	0.979(0.968,0.991)	< 0.001	0.979(0.968,0.990)	< 0.001		
log-SDR	0.939(0.921,0.959)	< 0.001	0.962(0.941,0.984)	< 0.001	0.975(0.957,0.994)	0.012	0.983(0.963,0.998)	0.048

Only variables retaining independent statistical significance (*p* < 0.05) in the final multivariable model are shown in the “multivariate analysis” column.

*SBP* systolic blood pressure, *NYHA* New York Heart Association, *WBC* white blood cell, *HGB* haemoglobin, *GLB* globulin, *UCB* unconjugated bilirubin, *cTn* cardiac troponin, *BNP* brain natriuretic peptide, *TC* total cholesterol, *eGFR* estimated glomerular filtration rate, *log-SDR* sodium to log(D-dimer) ratio

**Fig. 3:**
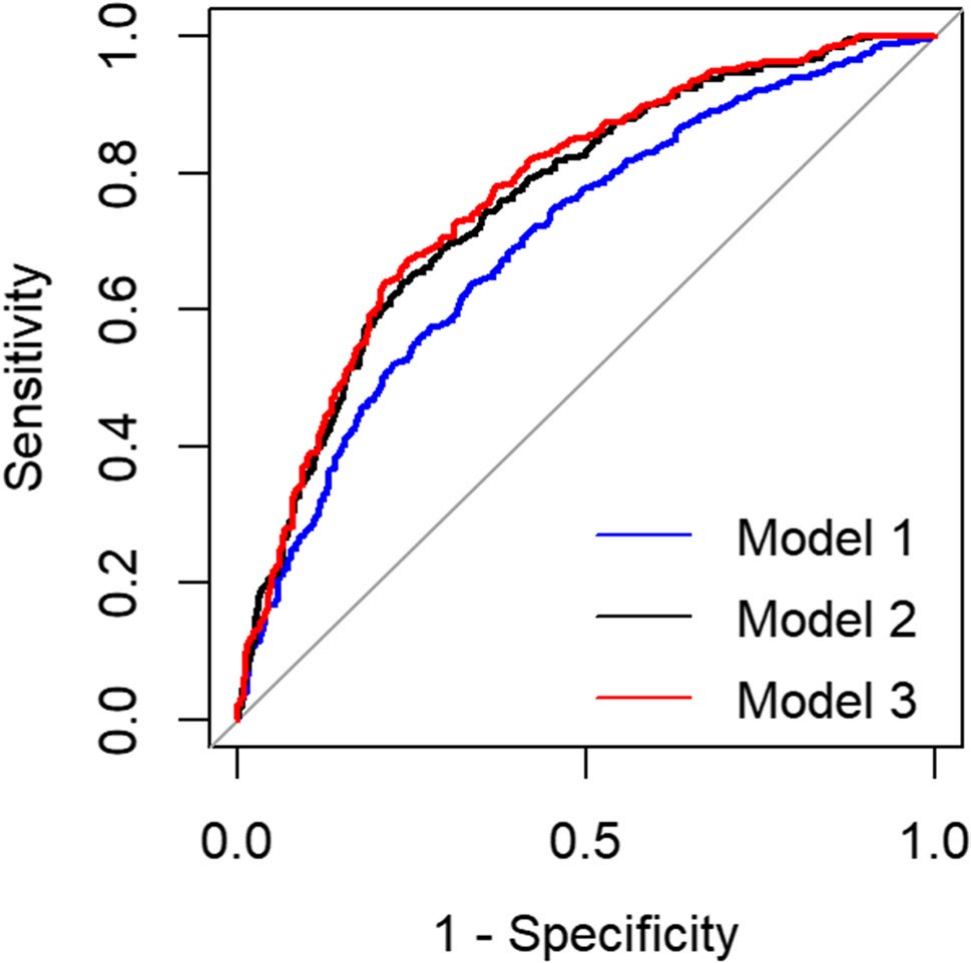
The ROC curves according to the three models’ predictive value for all patients. Model 1: adjusted for age, sex, BMI, DBP and NYHA. Model 2: adjusted for the variables included in model 1 and LogBNP, WBC, AST, GLB, Cr, BUN, potassium, Fib and LDL-C. Model 3: adjusted for the variables included in model 2 and log-SDR

To further assess the predictive value of log-SDR, three base models were constructed. Model 1 was adjusted for age, sex, body mass index (BMI), diastolic blood pressure (DBP), and NYHA class. Model 2 included all variables in Model 1 plus log-BNP, WBC, AST, GLB, creatinine (Cr), BUN, potassium, Fibrinogen(Fib), and low-density lipoprotein cholesterol (LDL-C). Model 3 further incorporated log-SDR into Model 2. The AUC for predicting CHF was 0.712 (*p* < 0.001) for Model 1, 0.752 (*p* < 0.001) for Model 2, and 0.781 (*p* < 0.001) for Model 3. Model 3, which included log-SDR, demonstrated the highest AUC compared with Models 1 and 2, indicating that log-SDR has good predictive value for the prognosis of CHF (Fig. 3) (Table 4).

**Table 4:** The predictive value of the log-SDR for all patients according to the three models

	AUC	*p*	Sensitivity	Specificity
Model 1	0.712	*P* < 0.001	67.7%	67.3%
Model 2	0.752	*P* < 0.001	76.2%	64.3%
Model 3	0.781	*P* < 0.001	75.7%	63.7%

DeLong’s test was used to compare the AUCs of correlated ROC curves: Model 1 vs. Model 3 *p* < 0.001 ; Model 2 vs. Model 3, *p* = 0.024

Model 1: Adjusted for age, sex, BMI, DBP and NYHA

Model 2: Adjusted for the variables included in Model 1 and LogBNP, WBC, AST, GLB, Cr, BUN, potassium, Fib and LDL-C

Model 3: Adjusted for the variables included in Model 2 and log-SDR

## Discussion

In this retrospective study, the prognostic effect of log-SDR on patients with different types of CHF in the clinical setting was explored. Our data indicate that patients with lower log-SDR values have a significantly higher risk of all-cause mortality compared to those with higher log-SDR values. Firstly, both patients with HFrEF and HFmrEF, as well as those with HFpEF, showed a significant correlation between higher log-SDR values and longer survival periods. Secondly, log-SDR can serve as an independent predictor for all CHF patients or for the subgroups of CHF including HFpEF and HFrEF plus HFmrEF. Even after multivariate adjustment for other factors (such as age, gender, SBP, NYHA cardiac function classification, etc.), log-SDR was independently associated with the mortality of different types of CHF. Each unit increase in log-SDR was associated with a greater reduction in mortality risk among patients with HFrEF plus HFmrEF(HR:0.949, 95% CI 0.932–0.967, *p* < 0.001) compared to those with HFpEF (HR:0.980, 95% CI 0.964–0.997, *p* = 0.021) Thirdly, log-SDR demonstrates moderate predictive ability for the prognosis of CHF. While established indicators such as NYHA class and Lg-BNP have been well-validated in predicting HF outcomes, the model incorporating log-SDR in our analysis exhibited improved discriminative performance (AUC = 0.781). These findings suggest that log-SDR may provide additional prognostic information regarding all-cause mortality in HF patients across varying EF ranges.

One noteworthy finding of our study is that the log-SDR may possess greater prognostic value in female patients with HFrEF plus HFmrEF. This sex-specific disparity likely stems from distinct physiological mechanisms in women. Specifically, estrogen exerts inhibitory effects on sympathetic nervous activity and the RAAS [15], thereby promoting superior volume and neuroendocrine homeostasis. As a result, a higher SDR value may more accurately reflect preserved volume-regulatory and compensatory capacity in female patients. Furthermore, estrogen confers protective benefits on endothelial function, mitigates inflammatory responses, and attenuates platelet aggregation, collectively contributing to lower baseline levels of inflammation and thrombotic activation in women with HF [16, 17]. Consistent with this, subgroup analyses in prior studies have indicated that guideline-recommended core pharmacotherapies may yield greater clinical benefits in female patients [18]. Thus, the distinctive physiological profile of women appears to synergize with established HF treatment strategies, jointly underpinning the sex-based prognostic differences observed in our investigation.

Hyponatremia, a common manifestation of dysregulated fluid and electrolyte balance, has a prevalence of 11% to 27% among HF patients [19]. Although dietary sodium restriction is frequently recommended for HF self-care, studies have not established an association between the extent of sodium reduction and clinical outcomes, showing no clear benefit or harm within 6, 12, or 24 months [20]. Conversely, overly stringent sodium restriction—compounded by poor appetite (inadequate intake) and high-dose diuretics (excessive loss)—can induce hypovolemic hyponatremia, which is associated with increased mortality risk. Furthermore, in HF patients with renal impairment, enhanced fluid reabsorption can lead to dilutional hyponatremia and fluid overload, further worsening the clinical condition and prognosis [6]. The pathogenesis of hyponatraemia in HF patients is multifactorial. In HF, reduced cardiac output leads to insufficient effective circulating blood volume, subsequently impairing renal function and decreasing glomerular filtration rate, thereby causing water and sodium retention [19]. Furthermore, hyponatremia directly stimulates the renin angiotensin aldosterone system (RAAS) and arginine vasopressin (AVP) activity, resulting in increased heart rate, vasoconstriction, and renal water reabsorption. This neuroendocrine overactivation exacerbates fluid retention, increases cardiac afterload and myocardial oxygen demand, thereby accelerating myocardial cell death and fibrosis [21]. Additionally, chronic inflammation in HF dysregulates sodium transporters and channels in renal tubular epithelial cells, further promoting sodium and water retention [7]. Diuresis remains the cornerstone of HF management. However, many diuretics can induce or worsen electrolyte imbalances. Notably, thiazide diuretics are a frequent cause of hyponatremia, as they act on the distal convoluted tubule to inhibit sodium-chloride cotransport [22]. In hyponatremic HF patients, extracellular hypotonicity drives water into cells, causing cellular edema. Severe cases can lead to acute water intoxication and cerebral edema [23, 24]. Importantly, rapid and systematic correction of hyponatremia has been shown to reduce all-cause mortality in this population.

D-dimer is a widely used biomarker indicative of activated coagulation and fibrinolysis. Beyond its roles in diagnosing venous thromboembolism and predicting stroke risk in atrial fibrillation, it serves as an independent predictor of mortality and fatal events in various cardiovascular diseases [25]. Research has demonstrated a correlation between elevated D-dimer levels and the poor prognosis of patients with HF [9, 11]. HF is frequently accompanied by coagulopathy and an elevated risk of thromboembolism, attributed to a disordered coagulation system and a prothrombotic state. Although the underlying mechanisms are not fully elucidated, the Virchow triad—comprising blood stasis, endothelial injury, and hypercoagulability—is often present in HF. These abnormalities likely arise from multiple factors, including cardiac structural changes, endothelial dysfunction, inflammation, neurohormonal activation, and arrhythmias [26, 27]. While hypercoagulability is often considered transient, long-term thrombotic risk in HF may also be driven by persistent factors such as endothelial injury, chronic inflammation, and hemodynamic abnormalities, in addition to reversible causes like immobility during hospitalization [28]. This heightened thrombotic risk can lead to severe complications like cardiogenic stroke, systemic embolism, and venous thromboembolism, significantly impairing patients’ quality of life and prognosis. The risk is particularly pronounced in HF patients with sinus rhythm and is further exacerbated by concomitant atrial fibrillation [29]. Moreover, elevated D-dimer levels may exacerbate the inflammatory response by promoting inflammatory cytokine release, thereby adding to the disease burden [30].

The aetiology of HF is multifactorial, and the resulting pathological changes during disease progression can vary [14]. In view of the divergent mechanisms of action of serum sodium and D-dimer in differing types of HF, it is imperative to ascertain the prognostic value of the log-SDR in various types of HF. HFrEF is characterised by a series of complex changes at the molecular and cellular levels of the heart, ultimately leading to a decline in cardiac pumping function [31]. This, in turn, causes a decrease in cardiac output, blood stasis in the ventricles, and thrombosis, and continuously activates the coagulation and fibrinolysis systems. The organism continuously activates the sympathetic nervous system (SNS) and the renin-angiotensin-aldosterone system (RAAS) in order to maintain homeostasis. This results in sodium and water retention, vascular contraction, exacerbation of fluid load, and further deterioration of cardiac function. HFpEF accounts for approximately 50% of patients diagnosed with HF [32]. The cardiac structure and function abnormalities associated with this condition differ from those observed in HFrEF, frequently manifesting as diastolic dysfunction and other symptoms [33]. At present, the diagnosis and treatment of HFpEF are lacking in standardisation, with the consequence that many patients are being misdiagnosed or not receiving any diagnosis at all. This may be accelerating the progression of the disease.

This study represents the first systematic investigation of the association between log-SDR and all-cause mortality in patients with HF, an association that remains consistent across the HF spectrum categorized by EF. The results suggest that log-SDR may effectively complement traditional prognostic indicators such as NYHA class and BNP levels, thereby refining risk stratification. A log-SDR value below 47.90 can serve as a preliminary threshold for identifying high-risk patients. These individuals typically demonstrate significant volume overload, pronounced inflammatory activity, and prothrombotic activation. Clinically, they present with higher NYHA class, elevated BNP levels, and frequently have comorbid conditions such as renal impairment. Consequently, their risks of rehospitalization, mortality, and thromboembolic events are substantially increased. For this population, intensifying clinical monitoring—for example, by shortening follow-up intervals—and optimizing treatment strategies should be considered. These may include titrating neurohormonal antagonists, adjusting diuretic therapy according to hemodynamic status, and implementing multidisciplinary care focused on volume management and inflammation modulation. Conversely, patients with elevated log-SDR values generally exhibit better-preserved cardiac output, minimal thrombosis-inflammatory activation, and more favorable clinical outcomes. Thus, stratifying HF phenotypes based on log-SDR could support the development of standardized, risk-adapted management pathways and facilitate early intervention.

## Limitations

This study has several limitations. First, the use of anticoagulant therapy was not systematically documented during data collection. Since anticoagulants can substantially reduce D-dimer levels by inhibiting coagulation cascade activation and fibrin degradation, this unmeasured confounder may have partially inflated log-SDR values, warranting cautious interpretation when evaluating log-SDR as a prognostic marker. Second, D-dimer levels are influenced by multiple factors, including infection, thrombosis, and malignancy. Although patients with known malignancies or severe hepatic or renal dysfunction were excluded, and relevant covariates were adjusted, residual confounding from unmeasured variables—such as subclinical thrombosis or occult infection—cannot be fully eliminated in an observational design. Third, the retrospective single-center design may introduce selection bias. As the cohort predominantly comprised patients with NYHA class III–IV HF, the generalizability of findings to all HF patients, especially those with milder symptoms, requires further verification. Fourth, serum sodium and D-dimer were measured only at admission and do not capture their longitudinal variability, which may limit the precision of prognostic evaluation. Furthermore, interpretation of log-SDR as a ratio-based metric may be less reliable in patients with very low baseline D-dimer levels.

In conclusion, before log-SDR can be adopted in clinical practice, it requires further validation and refinement through large-scale, multicenter prospective studies involving the full spectrum of HF patients.

## Conclusion

In summary, our findings indicate that log-SDR shows potential as a biomarker for long-term mortality risk stratification in CHF patients, independent of EF. Lower log-SDR levels were associated with increased all-cause mortality risk. The dynamic monitoring of log-SDR may contribute to refined risk assessment approaches; however, further validation in external cohorts is necessary before considering clinical implementation. With such validation, log-SDR could potentially support clinicians in optimizing individualized treatment strategies.

## Supplementary Information

Supplementary Material 1.

## Data Availability

The datasets used and/or analysed during the current study available from the corresponding author on reasonable request.
