# Supplementary material for: Predictive value of the serum sodium to log(D-dimer) ratio for the risk of all-cause death in patients with chronic heart failure with different ejection fractions
Source: BMC Cardiovasc Disord. 2026 Jul 20;26:613. doi: 10.1186/s12872-026-05609-y (PMC13383445; doi:10.1186/s12872-026-05609-y)

| **Supplementary Table 1.**Comparison of the Predictive Performance of log - SDR, Serum Sodium, and log(D - dimer) Using NRI and IDI | | | | | | |
| --- | --- | --- | --- | --- | --- | --- |
| Comparison Group | NRI | 95% CI for NRI | P - value for NRI | IDI | 95% CI for IDI | P - value for IDI |
| log - SDR vs Serum Sodium | 0.223 | 0.102–0.345 | <0.001 | 0.043 | 0.024–0.061 | <0.001 |
| log - SDR vs log(D - dimer) | 0.160 | 0.044–0.277 | 0.007 | 0.022 | 0.010–0.033 | 0.036 |
| Abbreviations: CI, confidence interval; IDI, integrated discrimination improvement; NRI, net reclassification improvement; log‑SDR, serum sodium‑to‑log(D‑dimer) ratio.  All models were adjusted for the same set of covariates: age, sex, body mass index(BMI), New York Heart Association functional class(NYHA class), log-transformed B-type natriuretic peptide(logBNP), white blood cell count(WBC), creatinine(Cr), blood urea nitrogen(BUN), potassium, fibrinogen(Fib), and low-density lipoprotein cholesterol(LDL-C). | | | | | | |

| **Supplementary Table 2.**Comparison of Cox regression models for all‑cause mortality using different core biomarkers | | | |
| --- | --- | --- | --- |
| Model and Core Variables | Adjusted HR (95% CI) | P-value | C-index (95% CI) |
| Model4:log-SDR | 0.964 (0.952,0.976) | <0.001 | 0.776(0.746,0.805) |
| Model5:Serum Sodium | 0.971 (0.949,0.993) | 0.009 | 0.765(0.734,0.795) |
| Model6:log(D - dimer) | 1.643 (1.215,1.973) | 0.001 | 0.773(0.743,0.803) |
| **Note**: All models are multivariable Cox proportional hazards models. The core variables (SDR, serum sodium, and log-transformed D-dimer) were analyzed as **continuous variables**. All models were adjusted for the same set of covariates: age, sex, body mass index(BMI), New York Heart Association functional class(NYHA class), log-transformed B-type natriuretic peptide(logBNP), white blood cell count(WBC), creatinine(Cr), blood urea nitrogen(BUN), potassium, fibrinogen(Fib), and low-density lipoprotein cholesterol(LDL-C). HRs correspond to a one‑unit increase in the respective continuous core variable. | | | |

**Supplementary Figure 1.** Patient enrollment flow chart


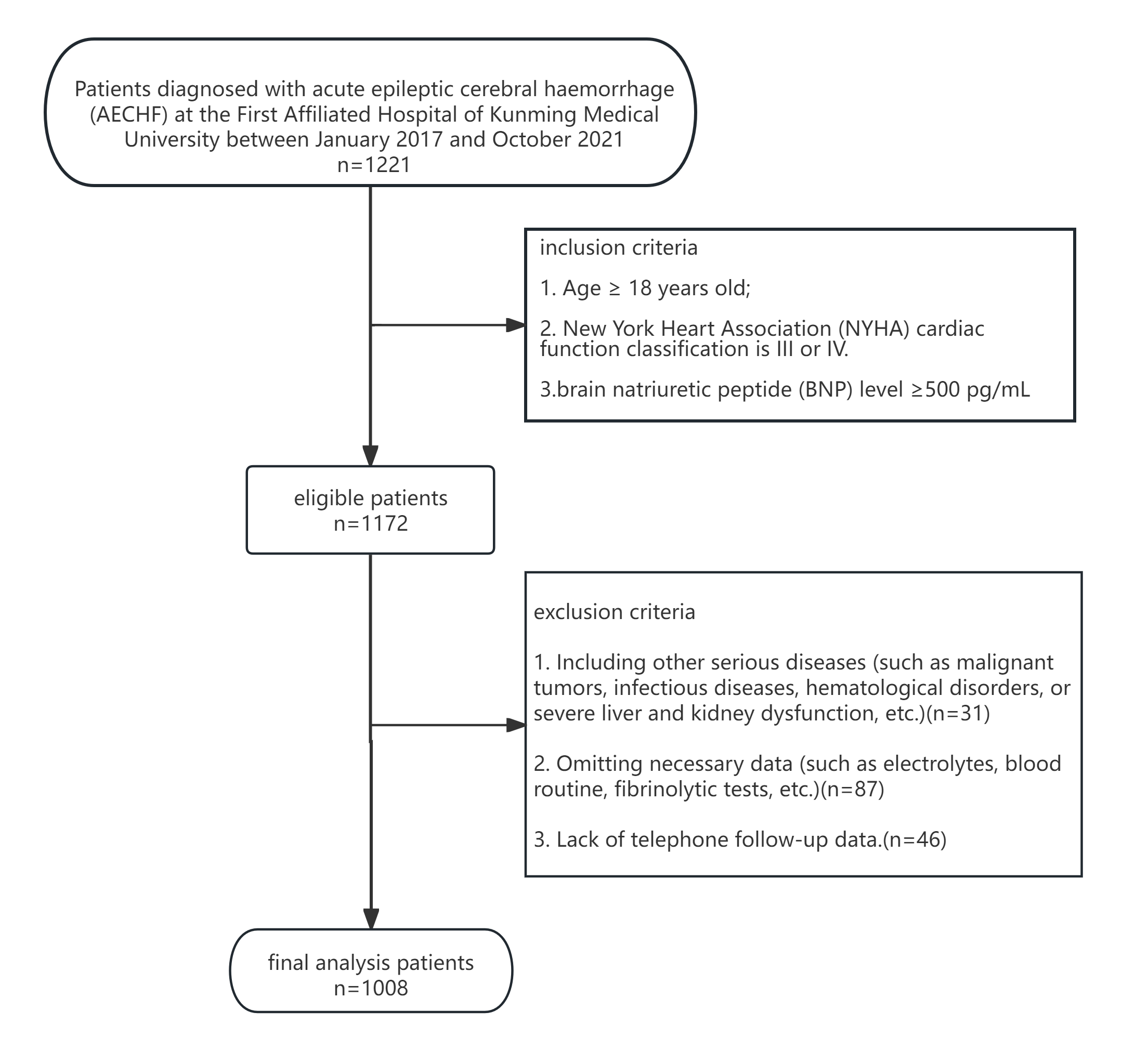

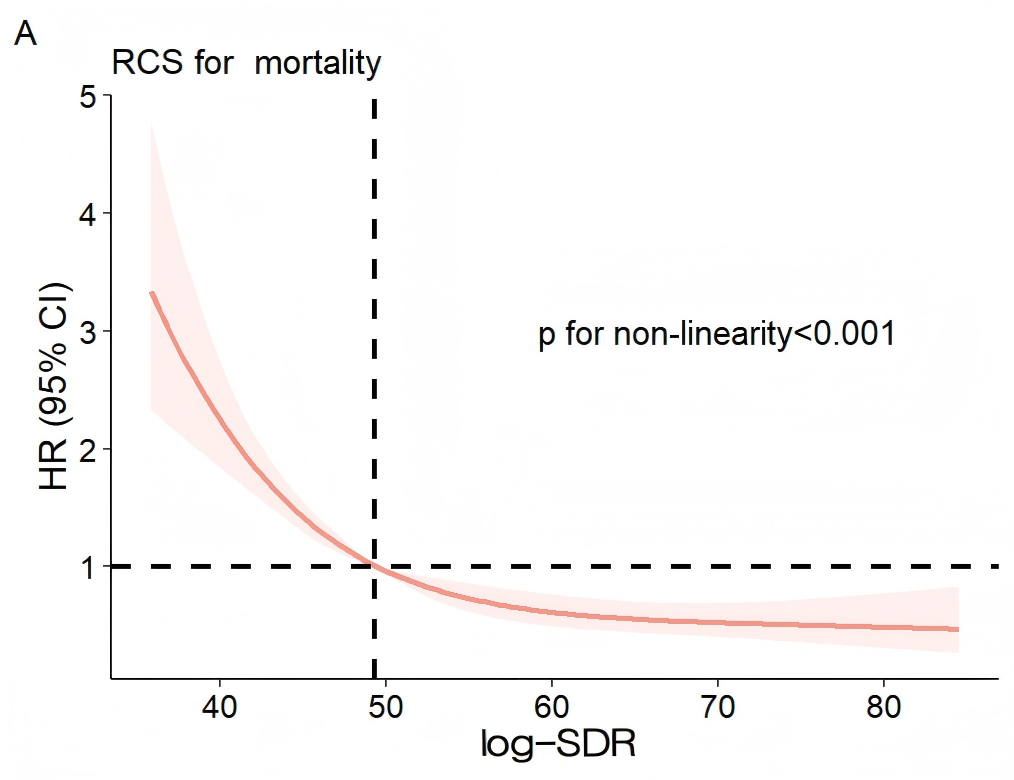


**Supplementary Figure 3**. Nonlinear relationship between log-SDR and all-cause mortality in all patients.

**Supplementary Figure 2** Box plot


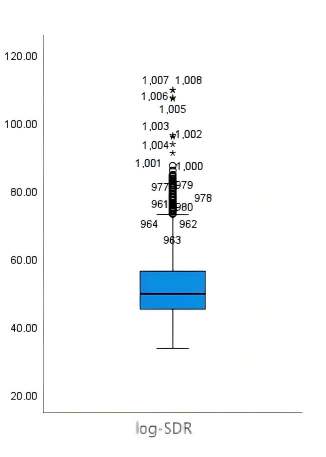

Supplement: Supplementary file 1 — Supplementary Material 1. [file 12872_2026_5609_MOESM1_ESM.docx]
